# Quantitative Mass Spectrometric Analysis of Autoantibodies as a Paradigm Shift in Autoimmune Serology

**DOI:** 10.3389/fimmu.2019.02845

**Published:** 2019-12-04

**Authors:** Adrian Y. S. Lee, Tim Chataway, Alex D. Colella, Tom P. Gordon, Jing J. Wang

**Affiliations:** ^1^Department of Immunology, SA Pathology, Flinders Medical Centre, Adelaide, SA, Australia; ^2^College of Medicine and Public Health, Flinders University, Adelaide, SA, Australia

**Keywords:** autoantibodies, autoimmunity, lupus, Sjögren's syndrome, rheumatoid factor, mass spectrometric sequencing

## Introduction

Affecting ~7–9% of individuals worldwide (1), autoimmunity is a relatively common condition that can cause substantial morbidity and mortality (2). However, there are considerable challenges in finding robust and accurate biomarkers for this heterogeneous group of diseases. Serum autoantibodies have served as archetypal diagnostic biomarkers for autoimmune diseases over decades (3). As pathologic species, they can be used to monitor disease activity and treatment responses (4).

Most diagnostic laboratory tests for autoantibodies utilize conventional assays such as the solid-phase enzyme immunoassay (EIA), enzyme-linked immunosorbent assay (ELISA), or radioimmunoassay (RIA). All of these assays quantitate amounts of autoantibodies in the bodily fluid but fail to delineate their molecular composition. Multiplex assays have emerged for autoantibody high-throughput screening that enable rapid identification of subsets of patients to facilitate diagnostic and predictive medicine (5). This is particularly important since multiple autoantibodies are often responsible for autoimmune disease (6). However, such conventional assays cannot unravel clonal evolution and dynamic autoimmune responses. Frustratingly, prediction of disease onset and flares with these biomarkers remains suboptimal.

Mass spectrometry (MS) is an analytical technique which can identify proteins by determining the amino acid sequence of peptides derived from each protein. MS can also measure changes in relative abundance of specific proteins as a consequence of treatment, and with appropriate standards, quantify absolute abundance. MS has been used previously to analyze specific antibodies or the repertoire of antibodies in order to better understand the dynamics of humoral immune responses in vaccinated animals (7).

This technology has been used to characterize autoantibodies in diseases such as systemic lupus erythematosus (SLE) and Sjögren's syndrome (SS) by identifying their immunoglobulin variable region (IgV) subfamily usage and mutational profiles at a molecular level (8). Despite conventional immunoassays determining stability in autoantibody profiles, MS-based quantitative proteomics has been used to uncover the dynamic changes in molecular signatures and levels of autoantibodies as the disease progresses (9). Subtle nuances in the molecular profile of patient autoantibodies can be identified, paving the way for new diagnostic biomarkers that can anticipate the onset or severity of disease before conventional biomarkers or immunoassays (10). This exciting technology hence offers a unique opportunity to identify pathogenic “rogue” and/or protective clonotypes that characterize autoimmune diseases. By deconstructing these clonotypes by quantitative proteomics and establishing a database of clonotypes with their corresponding pathogenicity, this would possibly facilitate identification of at-risk patients for deterioration, or predict response to targeted therapy.

## Quantitative Proteomics

### Workflow and Challenges of Quantitative Autoantibody Proteomics

**Figure 1:**
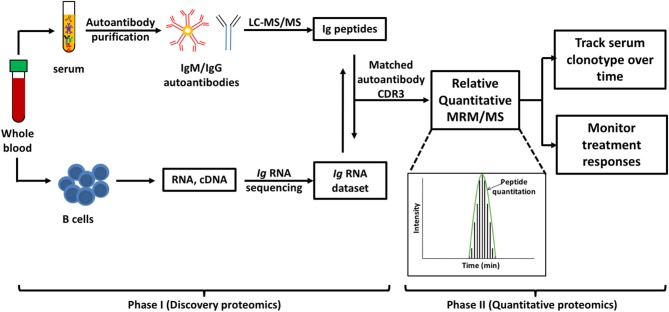
Workflow for quantitative autoantibody proteomics. Briefly, IgM or IgG autoantibodies are affinity purified from patient serum and sequenced by liquid chromatography mass spectrometry/mass spectrometry (LC-MS/MS). Ig variable region peptide sequences are searched against the matched Ig RNA dataset to identify clonotypic complementarity determining 3 regions (CDR3) peptides in the serum proteome (Discovery proteomics). These peptide “barcodes” are then used for relative quantitative multiple reaction monitoring (MRM)/MS platforms to quantify the specific clonotypes in longitudinal samples or following treatment (quantitative proteomics). Peptides of interest are monitored as as they elute from the HPLC and the level of each peptide in the samples is quantified based on the subsequent abundance chromatography curves.

**Figure 2:**
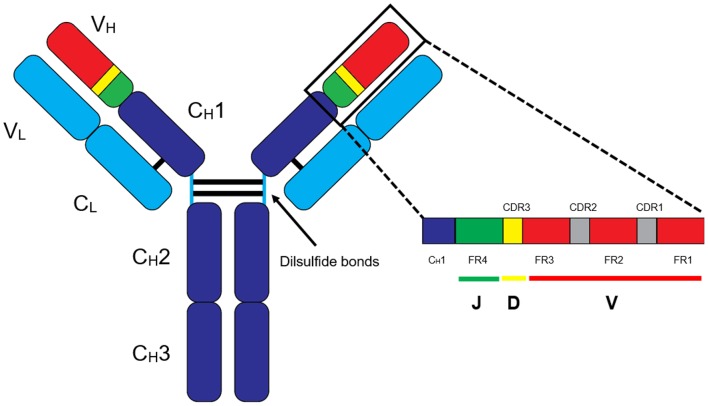
Basic structure of an IgG antibody. The IgG antibody is made out of variable (V) and constant (C) domains found in heavy (H) and light (L) chains. The variable-diversity-joining (VDJ) region is found in the heavy chain variable (V_H_) region, and VJ region is found in the light chain variable (V_L_) region. In general, clonotype “barcodes” are peptides from heavy chain third complementarity-determining regions (HCDR3) of the autoantibodies flanked by framework regions (FR).

MS-based autoantibody analysis workflow constitutes two phases (Figure 1). Discovery proteomics of IgV peptides is first performed by processing enzyme-digested purified autoantibodies on a highly accurate mass spectrometer such as a qTOF or Q Exactive. Several techniques exist to isolate antibodies, such as column-based affinity purification. Recently, agarose gel-based immunoprecipitation has been used which only requires microliters of fresh or archived serum (11). The MS spectra are analyzed by software such as PEAKS (Bioinformatics solution Inc., Ontario, Canada) which combines a *de novo* sequencing module (determining the amino acid sequence independent of a database) with a database matching module which aligns all amino acid sequences against a database of known antibodies such as the ImMunoGeneTics (IMGT) database. Heavy-chain third complementarity-determining regions (HCDR3s) are hypervariable and generate most of the diversity in the human antibody repertoire as well as being a major determinant of binding specificity. HCDR3s, along with details of the immunoglobulin heavy chain variable (IGHV) and joining (IGHJ) gene segments, define the clonotype of antibody, and peptides from HCDR3 region can serve as clonotypic markers of an antibody (12) (Figure 2). Currently, the lack of reference databases for rearranged variable-diversity-joining (VDJ) segments represents a considerable challenge for proteomic workflows for autoantibody sequencing. To solve this issue, in parallel to serum proteomics, IgH RNA nucleotide sequences are obtained from peripheral blood mononuclear cells (PBMCs) in the same patient and used as personalized VDJ segment reference libraries (Figure 1). The matched HCDR3 peptides can then serve as surrogate clonotypic markers of autoantibodies for clonotype tracking in the second phase of the workflow (Figure 1).

The second phase quantifies antibody clonotypes of interest (e.g., a pathogenic clone) by measuring the individual unique “barcodes” of relevant clonotypes from a single patient (Figure 1). This is performed using a technique called MRM (multiple reaction monitoring) (Figure 1). For expression profiling of human autoantibodies, a quantitative MRM/MS platform based on surrogate IgV subfamily and CDR3 peptides is adapted for targeted identification and monitoring of expression of pathogenic clonotypes in patient sera over time (11). These peptides are quantified in a multiplex platform that can potentially cover multiple clonal variants derived from linked sets of autoantibodies. Quantitative proteomics have been used to quantify HCDRs peptides following tetanus toxoid booster vaccination (13), to investigate vaccine-elicited antibody clonotypes before and after influenza vaccination (14) and to discover persisting antibodies by longitudinal profiling of serum anti-H1N1 antibodies (15). Relative quantification determines fold changes in the levels of clonotypic peptides from one time-point to another and compares only the same clonotypes. Accurate relative quantification requires identical processing and loading of samples, with each time point analyzed within a single batch. Absolute quantification can be performed by spiking samples with known quantities of identical peptides with incorporated stable isotopes. Although quantitation of clonotypes via HCDR3 sequencing is more helpful to track disease in an individual patient, quantification across different patients is theoretically possible but has not yet been explored in the scientific literature.

By isolating and purifying the autoantibodies of interest, MS analysis can resolve a molecule of interest at the amino acid level. Purifying specific autoantibodies, discovery MS, bioinformatics analysis followed by MRM relative quantification, takes ~2–3 days.

Although foreshadowed as a tool to analyze complex immunological systems (16), quantitative proteomics has not been translated until now to the emerging field of MS-based antibody proteomics. Here, we will examine recent practical applications of this technology for targeting two iconic blood autoantibodies: rheumatoid factors (RFs) in primary SS and anti-dsDNA in SLE. In this Opinion Piece, we will also explore how MS technology is starting to become integrated into the understanding of other autoimmune diseases.

### Rheumatoid Factors in Sjögren's Disease

RFs are autoantibodies directed against the Fc region of IgG, frequently of the IgM isotype. They are commonly found in rheumatoid arthritis, SS and SLE as well as chronic infections, interstitial lung disease and endocarditis (17). In primary SS, their presence is an independent predictive factor for the development of lymphomas which is thought to arise from chronic stimulation of RF-positive B cells (18). RFs may also precipitate as cryoglobulins and can cause devastating end-organ damage. Recently, quantitative proteomic technology distinguished the unique molecular profiles of cryoprecipitable RFs from the soluble RF in a group of primary SS patients (19) and in cryoglobulins (20). With time, RFs were shown to become more pathogenic as they accumulated mutations. This was made possible by the concurrent proteomic analysis of isolated serum RF IgM heavy chains and transcriptomic analysis of *IGH* RNA data from matched PBMCs. Shared HCDR3 sequences were found between unrelated patients indicating common elements to the pathogenicity of RFs. Moreover, pathogenic HCDR3 peptides were able to be detected in the serum years before the onset of detection of cryoglobulinemia by conventional assays or clinically apparent mixed cryoglobulinemia, whereas levels of pathogenic clonotypic peptides decreased following immunosuppression and remission of mixed cryoglobulinemia (19).

By extension, pathogenic and benign clones can also be tracked horizontally in time, providing a further dimension to the current, widely-adopted quantitative proteomics of disease biomarkers. Such resolution of molecular profiling may be useful in creating libraries of pathogenic clonotypes and therefore, predicting patients who may form serious cryoglobulinemic complications.

### Deconstructing Anti-dsDNA in SLE

Anti-dsDNA are the hallmark autoantibodies of SLE and have become incorporated in the diagnostic criteria for the disease. The antibodies have strong links with lupus nephritis and are correlated with disease activity (21). A variety of conventional assays have been used to detect these antibodies including the Farr radioimmunoassay, *Crithidia luciliae* immunofluorescence test (CLIFT), and ELISA—each of these techniques display unique diagnostic specificities and sensitivities, as well as technical limitations (22). The Farr and CLIFT assays detect higher affinity anti-dsDNA to native DNA than the ELISA. As a result, the CLIFT and Farr assays have high diagnostic specificities for SLE whilst the ELISA methods have higher (moderate) sensitivities (23, 24) raising the need to develop alternative approaches to profile subpopulations of these clinically important autoantibodies.

Recently, conserved and mutated regions of secreted high affinity anti-dsDNA IgV subfamily peptides and light-chain CDR3 clonotypic peptides have been analyzed in serial serum samples using quantitative MRM proteomics. For the first time, heavily mutated, pathogenic clonotypes can be tracked, quantified and parallel total anti-dsDNA levels (by Farr assay) using as little as 50 microliters of sera (11).

**Figure 3:**
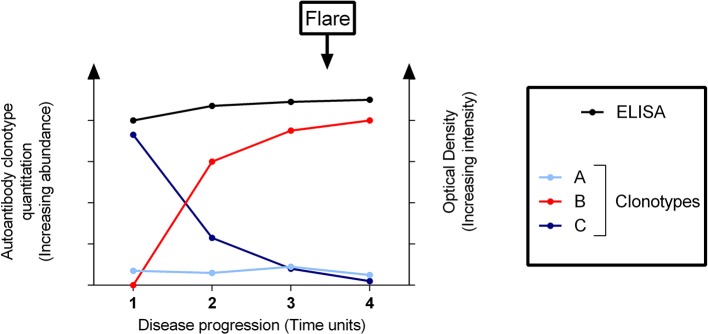
Clonotypic profiling of a pathogenic autoantibody predicts a flare of disease undetectable by solid-phase immunoassay. Conventional assays (e.g., enzyme-linked immunosorbent assay [ELISA]) cannot differentiate between different clonotypes–here designated as clonotypes A, B, and C as distinguished by quantitative proteomics–which comprise the total detectable autoantibodies. The disease flare is not predicted by the ELISA, whilst quantitative proteomic assays are able to detect the pathogenic clonotype B rising significantly before the onset of a flare. Clonotypes A and C are effectively “out-competed”.

In a similar manner to RF-mediated cryoglobulinemic vasculitis in SS, pathogenic anti-dsDNA clonotypes can potentially be detected by quantitative proteomics in the phase preceding SLE flares while masked by mixtures of other clonotypes using routine immunoassay (shown schematically as a theoretic model in Figure 3). Thus, quantitative proteomics may have clear advantages in profiling and tracking pathogenic autoantibody subsets compared with current tests of global autoantibody readouts. Similar to the detection of RF/cryoglobulins years before the onset of clinical manifestations (see “Rheumatoid factors in Sjögren's disease”), quantitative proteomics offers a more sensitive and accurate methodology for detecting pathogenic autoantibodies ahead of time and hence, predicting a flare (Figure 3).

### Other Autoimmune Diseases

MS-based autoantibody sequencing technology has been applied to other organ-specific autoimmune diseases. In celiac disease, MS has been used to deconstruct the molecular signatures of serum and gut transglutaminase IgA showing common V-region and HCDR3 elements; yet, with distinct compartment-specific differences (25, 26). These additional data provide insight into the pathogenesis of this disease and show that common plasma B cell clones give rise to gut and serum disease-specific IgA. Similarly, in the pemphigus group of blistering autoimmune skin diseases, desmoglein autoantibody repertoires have also been explored via MS, showing shared subfamily usage among patients (27). Interestingly, the authors also used discovery proteomics with customized software to determine relative quantitation of specific clonotypes and reported that individual circulating autoantibody clonotypes persisted over time (27).

Although there are only a few autoimmune diseases whereby their archetypal autoantibodies have been explored in detail by MS, this workflow is equally applicable to any other antibodies with high affinity and specificity that can be purified from body fluid or tissues, providing purified antigen is available. Therefore, great promise is in place to explore the wide range of iconic autoimmune diseases with characterized autoantibodies such as type 1 diabetes mellitus, and anti-neutrophil cytoplasmic antibody (ANCA) vasculitides. Whilst this technology is beginning to flourish as an exciting and powerful tool for biomarker discovery, very few studies to date have used it in autoantibody investigations, perhaps due to the challenges of dealing with a wide repertoire of autoantibodies. Even fewer studies have utilized the ability of MRM to provide a precise method of tracking each clonotypes as the disease unfolds. Indeed, further research is certainly needed to ascertain the degree of generalizability of the above results to the rest of the autoimmune diseases spectra.

## Challenges, Future Directions, and Conclusion

Matching MS data to transcript sequencing of B cells from the same patient significantly reduces the difficulty in identifying clonotypic HCDR3 sequences. However, the HCDR3 sequences of secreted autoantibodies might not be present in the reference BCR sequencing database which can occur if the antibody-secreting B cells reside in the bone marrow or target tissue and not in the sequenced peripheral blood. Where databases with complete rearranged VDJ segments are not available, *de novo* sequencing is employed which determines the amino acid sequence independent of a database. However, advanced expertise and extremely high-end accurate mass instrumentation is required for high confidence *de novo* sequencing of intact HCDR3 peptides.

The establishment of databases with clinically relevant and validated clonotypes (HCDR3 regions) is possible but will take considerable time and energy, especially with the processing and sequencing of an overwhelming number of key peptides. As of now, no such databases and definite clinical implications of clonotypes are not known. Furthermore, considering the massive diversity of antibodies, the creation of databases of antibody sequences to establish antibody specificity is not practical and is compounded by the fact that post-translational modification of sequences can dramatically alter antibody function and specificity.

A greater understanding of the secreted antibody repertoire in vaccine response (28) and infectious diseases both in the host and in the pathogenic entity (29) are some of the extended applications of this technology to other areas of medical science. Already, MS technology has become integrated into the diagnostic world to provide a multi-dimensional understanding of pathogens as they evolve from within the host (30), providing a plethora of useful information to clinicians and scientists. In addition, analysis of other bodily fluids, such as saliva and feces, compared to the serum proteome, may offer unique insights into the compartmentalization and microbiome that contributes to antibody repertoire and disease pathogenesis (31).

Complex autoimmune diseases are heterogeneous that have a vast range of clinical presentations, genetic, and molecular profiles, and hence, responses to treatment. We need to make a considered approach to identifying the unique molecular profiles of patients for diagnosis, treatment and risk stratification in order to develop personalized therapy (32). The arrival of proteomics has made it possible to characterize the complex antibody repertoire in diseases such as SLE (33), and quantitative proteomics extends the current capabilities of proteomic technology by allowing the tracking of dynamic protein changes in time and essentially zooming down onto these unique barcodes that signify their pathogenicity.

In summary, we argue that targeted MS is a unique technique with the potential to represent a paradigm shift in serological testing in autoimmune diseases. Further work, however, is desperately needed to explore its general applicability to a wider range of autoimmune diseases than presented here. It has an impressive multiplexing capacity for characterizing autoantibody IgV clonotypic peptides that have diagnostic and predictive potential at the proteomic level. Quantitation of such can be used to monitor disease activity, treatment responses and offer a new dimension of information above and beyond what modern day immunoassays can offer. In this exciting “omics” era, medicine now has an emerging tool to provide clinicians, medical scientists and patients a wealth of information, and continued exploration in this area will potentially see this integrated into routine clinical care in the future.

## Author Contributions

AL, TG, and JW conceptualized the paper, drafted and revised the manuscript. TC and AC substantively revised the manuscript. All authors approved the final version to be published, agreed both to be personally accountable for the author's own contributions and to ensure that questions related to the accuracy or integrity of any part of the work are appropriately investigated, resolved, and the resolution documented in the literature.

### Conflict of Interest

The authors declare that the research was conducted in the absence of any commercial or financial relationships that could be construed as a potential conflict of interest.
